# Carbon Fiber Epoxy Composites for Both Strengthening and Health Monitoring of Structures

**DOI:** 10.3390/s150510753

**Published:** 2015-05-06

**Authors:** Rita Salvado, Catarina Lopes, Leszek Szojda, Pedro Araújo, Marcin Gorski, Fernando José Velez, João Castro-Gomes, Rafal Krzywon

**Affiliations:** 1 FibEnTech—Research Unit of Fiber Materials and Environmental Technologies, Universidade da Beira Interior, 6201-001 Covilhã, Portugal; E-Mail: catarina.lopes@ubi.pt; 2 Department of Structural Engineering, Silesian University of Technology, ul. Akademicka 5, 44-100 Gliwice, Poland; E-Mails: Leszek.Szojda@polsl.pl (L.S.); Marcin.Gorski@polsl.pl (M.G.); Rafal.Krzywon@polsl.pl (R.K.); 3 Departamento de Informática, Instituto de Telecomunicações, Universidade da Beira Interior, 6201-001 Covilhã, Portugal; E-Mail: paraujo@ubi.pt; 4 Departamento de Engenharia Eletromecânica, Instituto de Telecomunicações, Universidade da Beira Interior, 6201-001 Covilhã, Portugal; E-Mail: fjv@ubi.pt; 5 C-MADE, Centre of Materials and Building Technologies, Departamento de Engenharia Civil e Arquitetura, Universidade da Beira Interior, 6201-001 Covilhã, Portugal; E-Mail: castro.gomes@ubi.pt

**Keywords:** structural health monitoring, carbon fibers polymer-matrix composites, self-sensing, strain sensors, electrical resistance

## Abstract

This paper presents a study of the electrical and mechanical behavior of several continuous carbon fibers epoxy composites for both strengthening and monitoring of structures. In these composites, the arrangement of fibers was deliberately diversified to test and understand the ability of the composites for self-sensing low strains. Composites with different arrangements of fibers and textile weaves, mainly unidirectional continuous carbon reinforced composites, were tested at the dynamometer. A two-probe method was considered to measure the relative electrical resistance of these composites during loading. The measured relative electrical resistance includes volume and contact electrical resistances. For all tested specimens, it increases with an increase in tensile strain, at low strain values. This is explained by the improved alignment of fibers and resulting reduction of the number of possible contacts between fibers during loading, increasing as a consequence the contact electrical resistance of the composite. Laboratory tests on strengthening of structural elements were also performed, making hand-made composites by the “wet process”, which is commonly used in civil engineering for the strengthening of all types of structures *in-situ*. Results show that the woven epoxy composite, used for strengthening of concrete elements is also able to sense low deformations, below 1%. Moreover, results clearly show that this textile sensor also improves the mechanical work of the strengthened structural elements, increasing their bearing capacity. Finally, the set of obtained results supports the concept of a textile fabric capable of both structural upgrade and self-monitoring of structures, especially large structures of difficult access and needing constant, sometimes very expensive, health monitoring.

## 1. Introduction

Carbon materials are important structural materials in aircraft, transport, construction, communication and lighting [1]. High-performance properties and cost-effective characteristics of carbon fibers enhance these and other emergent applications. In construction, some traditional methods for strengthening buildings are based on polymer matrix composites made of high performance fibers, such as continuous carbon fibers and carbon chopped fibers. These strengthening systems, such as FRP (Fiber Reinforced Polymer) and TMR (Textile Masonry Reinforcement), might integrate sensors for the structural health monitoring of the integrity of the structure of the building. Several techniques have been proposed for this monitoring [2,3,4] based on diverse types of sensors such as piezoresistive sensors [5,6] and optical fibers [7]. This paper focuses on the self-sensing ability of continuous carbon fibers to sense strain and damage. Indeed, as carbon fibers and their polymer-matrix composites are electrically conductive materials, they may function themselves as sensor of strain by considering the relationships between their electrical and mechanical behavior [1,8,9,10,11,12,13,14,15]. The self-sensing technique is based on changes in electrical resistance caused by strain and also by disruptions of the conductive pathway within the structure of the sensing material [13,15,16]. The self-sensing technique has been mainly studied for carbon fiber reinforced polymer (CFRP) laminated strips. However, this paper focuses on continuous carbon fibers reinforced composites with arrangements, mainly unidirectional, that may be in the future integrated into textile fabrics with ability for both strengthening and self-sensing functions. Indeed, textile weaving technology allows an easy control of the conductive pathways within the fabric by controlling the positioning of the sensing continuous fibers. Hence, textile technology might produce efficient and cheap strain sensors for monitoring systems. So, it is important to test the electromechanical behavior of continuous carbon fibers polymer-matrix composites. Moreover, this paper presents a study of the electrical resistance under low strain. In fact, in structural monitoring, low strain represents damage in the structure that is not visually observed and is, therefore, a structural health monitoring parameter of importance. In this paper, based on several previous studies [8,16,17], the monitoring of strain by electrical resistance has been based in two types of measurement: the through-thickness conductivity and the longitudinal conductivity (along the continuous fibers direction) measurement. The former gives information on the number of contacts between fibers in the plane of the material while the latter one gives information on the strain and fracture of the fibers. Therefore, they have also been named contact electrical resistance and volume electrical resistance, respectively. In general, one observes an increase in the relative electrical resistance with an increase of tensile strain. This occurrence is mainly due to an increase in the alignment of the fibers that reduces the number of possible contacts between fibers during loading [1,8,9,15,16,17,18].

This paper presents a preliminary study of the electrical and mechanical behavior of several continuous carbon fibers epoxy composites for strengthening of structures, whose geometry and arrangement of fibers were deliberately diversified to test and understand the ability of the strengthening composites for self-sensing low strains. The composites with different arrangement of fibers present a large range of initial electrical resistance values, to facilitate the study of how such arrangements can enhance the self-sensing ability. Plain weave woven fabrics were then produced to reinforce composites with self-sensing ability. Tensile tests at the dynamometer and laboratory tests on strengthening structural elements were also performed, making hand-made composites by the “wet process” *in-situ*, which is commonly used in civil engineering for the strengthening of all types of structures. These tests aim to analyze if such commonly practiced solution for external reinforcement of existing structures may allow self-monitoring of the strengthened structural element. Loading tests of a structural element on natural scale, a beam, enabled to calibrate the prototype of the textile sensor and verify its efficiency for strengthening. The obtained results support the definition of some guidelines for the development of textile fabrics capable of both strengthening and self-monitoring the health of structures.

## 2. Experimental Section

### 2.1. Materials and Specimens

#### 2.1.1. Carbon Tow

Tows of polyacrylonitrile (PAN) based continuous carbon fibers (filaments) of enhanced modulus were used to produce specific polymer-matrix composites. Table 1 presents their properties.

**Table 1:** Properties of the considered carbon tow.

Number of Filaments	Fineness of Tow (tex)	Tensile Strength (GPa)	Tensile Modulus (GPa)	Elongation at Break (%)	Filament Diameter (µm)	Density (g/m^3^)	Single Filament Resistivity (µΩm)
50 k	3200	4.0	253	1.6	7	1.80	15
24 k	1600	5.0	270	1.9	7	1.81	14

#### 2.1.2. Carbon Fiber Epoxy-Matrix Composite Probes for Preliminary Tests at the Dynamometer

In order to preliminary test the self-monitoring ability of carbon fiber epoxy-matrix composites, diverse specimens of composite materials were produced. Both used materials, the continuous carbon fibers and epoxy resin (S&P Resin 55), are materials commonly used for strengthening of structures in civil engineering. The probes, to be tested in the dynamometer, were thus produced at room temperature (around 18 °C 54% RH) through the following construction process:

- a first layer of epoxy-resin is spread on the top of an acrylic plate, which has a very smooth surface;
- then, carbon fibers are laid on this first layer of resin according to determined arrangements;
- further, another layer of resin is applied on the top of the fibers, embedding them very well;
- finally, the specimens are let to cure the resin for some days.

The dimensions of the produced specimens are presented in Table 2. The use of acrylic plates as supporting surface simplifies the removal of the probes, before testing them at the dynamometer. The design of the produced specimens is based on three assumptions. First, low strain (up to 1%) causes changes on the longitudinal electrical resistance. Second, a few intentional cuts along the length of filaments, although random, cause disruptions on the conductive pathway that highlight the change of the longitudinal electrical resistance. Third, intentional designs based on crossing continuous fibers enhance the contact electrical resistance.

**Table 2:** General features of specimens for preliminary tests at the Dynamometer.

**Carbon Filament Tow in Epoxy-Resin with 3 Days of Cure, with the Dimension of 700 × 50 mm.**	
A:Thin tow, with <50 k filaments,	B: solid thin tow, with <50 k and with eight partial cuts,
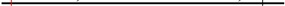	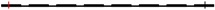
(*R*_0_ = 74.8 Ω)	(*R*_0_ = 100.5 Ω)
C: solid thin tow over thin tow, <50 k/<50 k,	D: solid thin tow, with <50 k, long length (snake design), 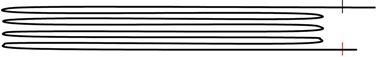 (*R*_0_ = 347.5 Ω)
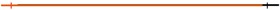	
(*R*_0_ = 84.4 Ω)	
E: Tow with 50 k and with six partial cuts,	
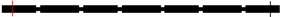	
(*R*_0_ = 8.6 Ω)	
**Carbon Filament Tow in Epoxy-Resin with 7 Days of Cure, with the Dimensions of 902 × 50 mm.**	
F: Tow over tow, 50 k/50 k,	G: solid thin tow over thin tow, <50 k/<50 k,
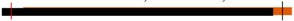	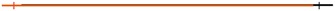
(*R*_0_ = 8.0 Ω)	(*R*_0_ = 63.1 Ω)
H: solid thin tow, with <50 k and with eight partial cuts,	I: solid thin tow over thin tow, <50 k/<50 k (snake over straight),
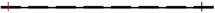	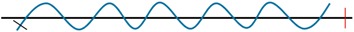
(*R*_0_ = 120.1 Ω)	(*R*_0_ = 72.2 Ω)

Table 2 presents the summary of the general characteristics of the diverse specimens that were made and tested at the dynamometer, including the initial electrical resistance R_0_. Different arrangements of reinforcement fibers were considered. Specimens A and D enable to check if low strains cause changes on the longitudinal electrical resistance of the composite, because the continuous carbon fiber provides a linear conductive path through the composite. Specimens B, E and H enable us to check if disruptions introduced in the conductive path, by making partial cuts along the tow, enhance the changes on the longitudinal electrical resistance of the composite. The disruptions are cuts made along the length of some filaments of the tow. Specimens C, F, G and I facilitate to check if intentional arrangements created by superposing two probes, which in the future may be easily integrated into a textile fabric, enhance the changes on the electrical resistance of the composite by increasing its contact electrical resistance. In these specimens, each of the two probes for measuring the electrical resistance is connected at one end of each of the two superposed tows, as shown by dark and light traces in the draws presented in Table 2. Specimens B and H, as well as specimens C and G, have resembling arrangement but different days of cure, helping to understand the change of the electrical resistivity as a function of the freedom of the filaments to move inside the composite.

#### 2.1.3. Woven Epoxy-Matrix Composite Probes for Preliminary Tests at the Dynamometer

**Figure 1:**
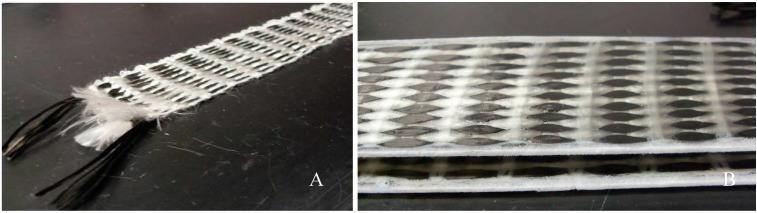
Images of a textile sensor (**A**) and of a woven epoxy composite probe (**B**).

Composites reinforced with woven fabrics were produced, assuring good infiltration of the tow by the resin. The woven substance is a hand-made fabric, plain weave, made of carbon tow with 24 k continuous fibers (density: 1.81 g/cm^3^), plus white acrylic (PAN) continuous fibers (density: 1.17 g/cm^3^) sewn together by a cotton yarn (density: 1.54 g/cm^3^). The fiber arrangement is mainly unidirectional, as shown in Figure 1. The woven substance, which was 5 × 45 cm^2^, was laid up in an open mold with the uncured resin (density: 1.11 kg/L). The probes were prepared by putting a smooth PTFE layer on each surface to improve the surface quality of the composites. The composites were under 50 bar compressive pressure for 24 h and were cured for seven days at room temperature.

The volume fractions of carbon fibers and of total fiber amount (carbon + acrylic + cotton) are presented in Table 3, as well as the thickness (average and standard deviation values of 10 measurements). The initial resistance is the average value of 60 measurements made every minute over one hour.

### 2.2. Tensile-Strain Tests While Recording the Electrical Resistance

Tensile tests at the dynamometer (Adamel Lhomargy DY35) were performed and the electrical resistance of the specimen was simultaneously recorded with a multimeter (Keithley 197A). The main purpose of these specific tests is the verification of the carbon fibers’ self-monitoring feature. Therefore, the performed tests were specifically adjusted to the experimental characterization of both electro and mechanical properties. The initial length was 700 mm for specimens A to D (3 days of cure), 902 mm for specimens F to I (7 days of cure) and 400 mm for probes 1 and 2. The specimens were pulled at 1 mm/min of velocity up to small displacements. The electrical resistance was recorded by a two-probe method, connecting the multimeter to the extremities of the specimen, which were previously attached with a pressed spring, in order to minimize the parasitic contact resistance. The relative variation of the electrical resistance (∆*R/R*_0_, specified in percentage) measured by the two-probe method includes the volume electrical resistance of the carbon filaments, the contact electrical resistance between the filaments and the parasitic contact resistance of the probe connections. As reported by several authors, if precautions are taken such as, for instance, covering the entire cross section of fibers with silver paint coating [9,15,17], this parasitic contact resistance may be minimized and become negligible. In these tests, it was minimized by attaching the extremities of the carbon tow with a pressed metallic clamp.

**Table 3:** General features of woven reinforced composite probes.

Probes	Type	Thickness (cm)	Carbon Fiber Volume Fraction	Total Fiber Volume Fraction	Initial Resistance (Ω)
1	24 k carbon fibers	0.17 ± 0.048	0.12	0.32	31.9 ± 0.01
	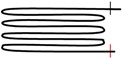				
2	24 k/24 k carbon fibers	0.17 ± 0.036	0.23	0.42	11.0 ± 0.03
	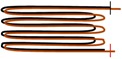				

### 2.3. Flexural Loading Tests of Concrete Elements for Bending Moment Analysis

**Figure 2:**
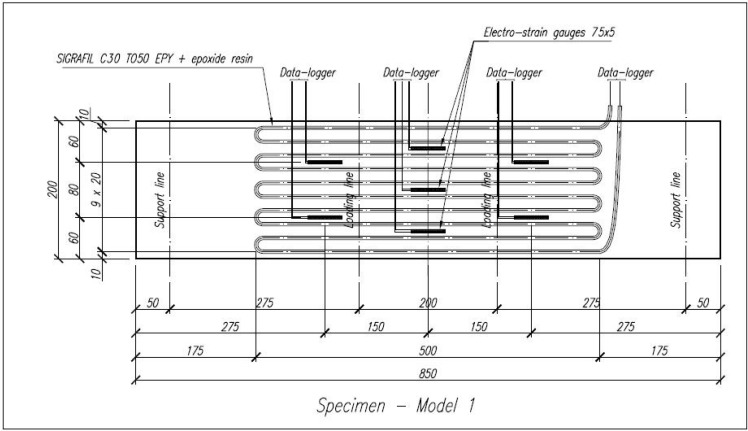
Scheme of specimens SP1 and SP2—strengthened with Continuous Carbon Fibers Epoxy Composite, with dimensions in mm.

Preliminary laboratory tests were performed, aiming to check if a common strengthening solution with Carbon Fibers Epoxy Composite may allow self-monitoring of the structural element by sensing low deformations. Four specimens, C16/20 concrete slabs with general dimensions 850 × 200 × 50 mm, were prepared. Specimens were cured in 20 °C temperature and 98% RH controlled room-curing conditions for 28 days prior to testing. Two specimens were considered as reference elements (SP3 and SP4) and two other specimens were strengthened with Continuous Carbon Fibers Epoxy Composite (SP1 and SP2), following general principles of structural design and civil engineering. The strengthening is based on a tow of 50 k filaments and around 7 m long, with similar arrangement to the snake design of specimen D previously presented in Table 2. Figure 2 shows the scheme of the reinforced concrete specimens of slabs SP1 and SP2.

**Figure 3:**
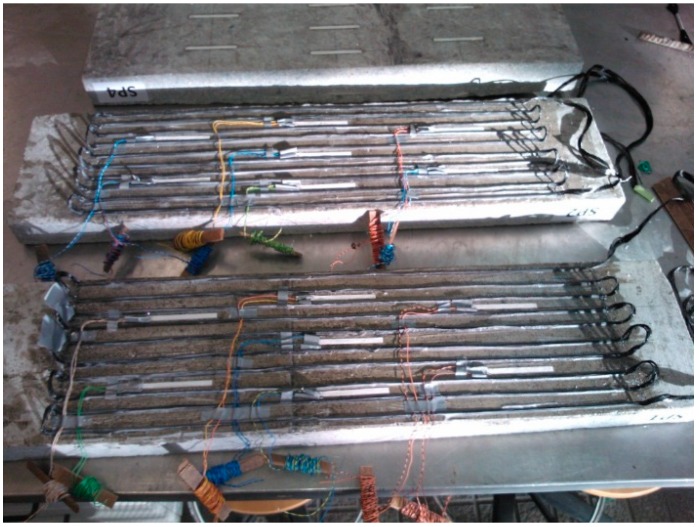
Bottom view of specimens SP1 and SP2—reinforced with Continuous Carbon Fibers Epoxy Composite.

The strengthening composite was prepared at room temperature (around 18 °C and 54% RH) using previously presented carbon fibers and epoxy resin (S&P Resin 55) according to the “wet process” *in-situ*, commonly practiced in civil engineering for strengthening of all type of structures. The surface of the concrete elements was previously cleaned with steel brush and stone disc, assuring the surface quality demanded by producers of FRP strengthening systems. All specimens were equipped with strain gauges, which served as reference for the measurement of the deformation of the concrete specimens. Figure 3 shows the view of the bottom surface of specimen with the strengthening continuous carbon fibers composite and the reference gauges.

**Figure 4:**
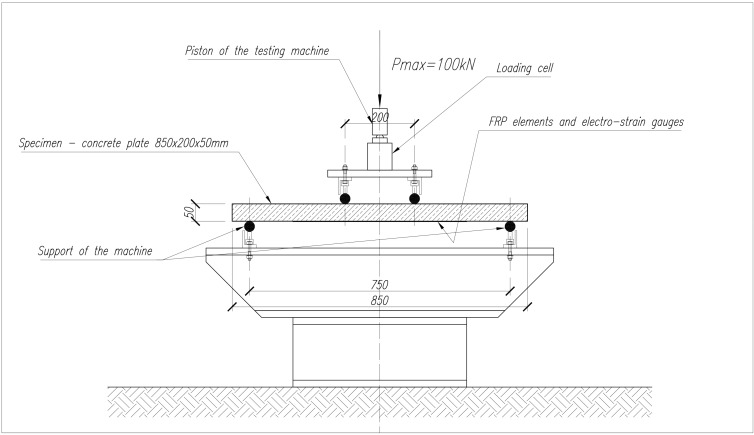
Scheme of the laboratory flexural strength-loading test of concrete slabs, with dimensions in mm.

Each of the four specimens (850 × 200 × 50 mm^3^) was tested according to the scheme of Figure 4, facilitating to verify the carbon fibers self-monitoring ability while changing load and deformation. The tests were performed slowly increasing the load (testing rate of 0.1 kN/s) and making a short break after each 5 kN load increase to stabilize the measured data of the relative electrical resistance of the carbon tow. During all tests, the results of strain-gauges as well as of the applying load were supervised and saved on Data-Logger and the variation of the electrical resistance of carbon tows were recorded by a two-probe method. The relative variation of the electrical resistance (∆*R/R*_0_, specified in %) measured by the two-probe method includes volume electrical resistance of the carbon filaments, contact electrical resistance between the filaments and parasitic contact resistance of the two probe connections. As previously, the parasitic one was minimized by attaching the extremities of the filaments with a pressed metallic clamp and by immobilizing the connections in the experimental installation.

### 2.4. Loading Tests of Reinforced Concrete Beam Subjected to Flexure

**Figure 5:**
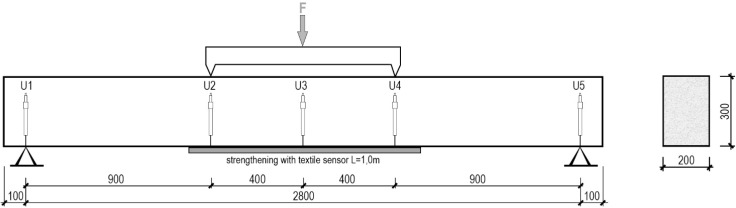
Scheme of the laboratory stand for loading test of reinforced concrete (RC) beams strengthened with textile sensor.

The concept of the textile sensor presented in this paper enables to simultaneously strengthen and monitor large structures, mainly reinforced concrete (RC) structures of difficult access as for example bridges. Thus, loading tests of a structural element in natural scale were performed to check the efficiency of the proposed solution in a final application. For this purpose, a self-monitoring and strengthening fabric was tested on a RC beam. The beam was loaded in a four-point bending test with a constant moment region of 800 mm, as shown in Figure 5. The textile fabric, 150 mm wide and 1000 mm long, was applied on the bottom surface along the length of constant bending moment.

The textile sensor is a hand made plain weave fabric, made of two 18 m-lengths of carbon tow with 24 k continuous fibers, similar to the fabric previously shown in Figure 1. The carbon fiber arrangement is mainly unidirectional. The two tows are superposed with a similar design to the weave that integrates probe 2, shown at Table 3. The electrical resistance of this textile sensor is 165.4 Ω, measured by connecting each of the two measuring probes to one end of each superposed tow.

During the test, the beam was monotonically loaded until failure. The load was applied using a hydraulic actuator and the test was carried out under displacement and strain control. In order to measure strain profiles along the tested textile, nine strain gauges were applied to the bottom surface over the textile sensor. Deflection of the beam was measured with linear variable displacement transducers (LVDT) (U1 to U5), as shown in Figure 5.

In addition to the most important goal of this test, which is the calibration of the textile sensor readings, the strengthening effect was also checked. For this purpose, the tested beam was compared to an identical beam with no strengthening and to an identical beam with commercial CFRP (Carbon Fiber Reinforced Polymer) solution, in the form of two layers of CFRP sheets.

## 3. Results and Discussion

### 3.1. Initial Electrical Resistance

Table 2 (which can be seen above) shows the initial electrical resistance for all fibers’ composite arrangements. The large range of magnitude of the values of the electrical resistance results from the consideration of the three hypotheses previously formulated in Paragraph 2.1.3. As reported by other authors, the arrangement of fibers in the composite may enhance the self-sensing function by augmenting changes in the electrical resistance that are too small to be detected [8,15,16]. As expected, thicker tows (specimens E and F) present lower electrical resistance, as they present a larger cross section of the conductive path than the thinner tows (specimens A and H). As well as a longer tow, specimen D presents higher electrical resistance than specimen A, which has a shorter tow, as expected. Disruptions introduced in the conductive path, by making a few partial cuts along the tow, increase the electrical resistance by providing a less linear conductive path through the composite, as anticipated. Moreover, the superposition of tows increases the electrical resistance, as can be observed by comparing values of specimen G and I. However, specimen A presents higher resistance than specimen G, which is probably due to the thinner cross section of its tow.

### 3.2. Tensile-Strain Tests at the Dynamometer

**Figure 6:**
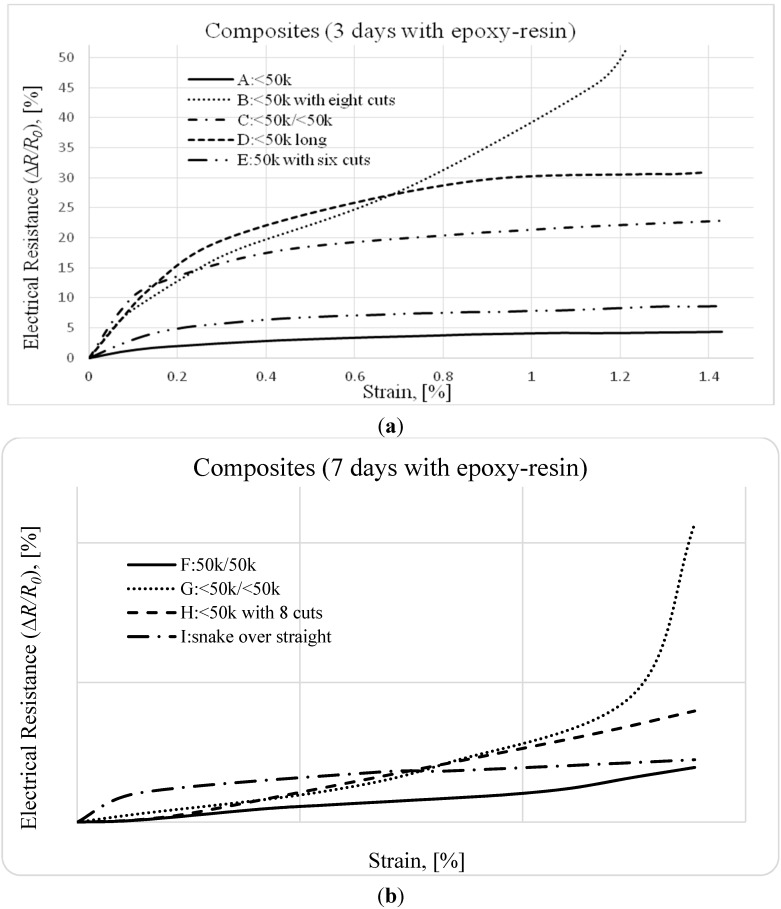
The relative electrical resistance *versus* strain of Continuous Carbon Fibers Epoxy Composites: (**a**) 3 days cure and (**b**) 7 days cure.

Figure 6 presents a relative increase in electrical resistance (∆*R/R*_0_), in percent, as a function of the strain. The measured electrical resistance includes both volume and contact electrical resistances. The contact resistance of the connections between the measuring probes and fibers, which was consistently minimized during the experiments, is assumed negligible.

As observed in Figure 6, for all tested specimens, the small strains cause an increase in the relative electrical resistance. This has been reported by other authors and explained by an increase in the alignment of the fibers, which reduces the number of possible contacts between fibers during loading [9,13,15,16,17,18]. This separation of the fibers, which are covered with the epoxy-resin, increases the electrical resistance of the composite.

In general, for unidirectional continuous carbon fibers in epoxy laminates under tensile loading, one observes an increase in the relative electrical resistance with an increase in tensile strain [1,8,9,15,16,17,18]. Corroborating this behavior, the results presented in this work show that the relative electrical resistance, which includes volume and contact electrical resistances, increases with an increase in tensile strain at low strain values.

This behavior is highlighted in this paper by the results obtained for the samples where the resin is not completely cured. In this case, the fibers have more freedom to move and become straighter than when the resin is fully cured, enhancing the effect of the alignment of the fibers on the increase of the contact electrical resistance, as reported in [8,16]. Although preliminary results for specimens B and G show an inadequate consolidation of the composite, caused by the uncured resin, they give guidelines for the design of a textile fabric that is able to both strengthen and monitor structures in concrete.

The initial electrical resistance, *R*_0_, of these 700 mm and 902 mm long specimens was deliberately diverse, varying from 8 Ω (for specimen F: thick tow over thick tow) to almost 350 Ω (for specimen D: solid thin tow with long length). Despite this large range of magnitude for the electrical resistance, in all specimens the relative change of the electrical resistance, ∆*R/R*_0_, was sensing the deformation. However, as expected, the results of the longitudinal electrical resistance of specimens A (<50 k) and D (<50 k long) demonstrate that higher initial electrical resistances enhance this sensing ability by increasing the precision of the measurement.

Moreover, the results of the specimens in which the contact electrical resistance was enhanced by superposing two tows, such as specimens C (<50 k/<50 k), F (50 k/50 k) and I (snake/<50 k), might improve the ability for self-monitoring of the strain. Additionally, this arrangement of unidirectional fibers, of two superposed tows, might be easily reproduced in a plain textile woven fabric.

Finally, by comparing the preliminary results obtained with specimens E (thick tow 50 k with 6 cuts), B (thin tow with 8 cuts) and H (thin tow with 8 cuts) with the ones obtained with specimen A (thin tow), one may conclude, that the introduction of deliberated disruptions on the conductive pathway, by random cuts, enhanced the sensing ability. However, as the number of filaments of the tow that are cut is unknown and insufficiently controlled, this option of arrangement was discarded when producing the prototypes of sensing fabric.

**Figure 7:**
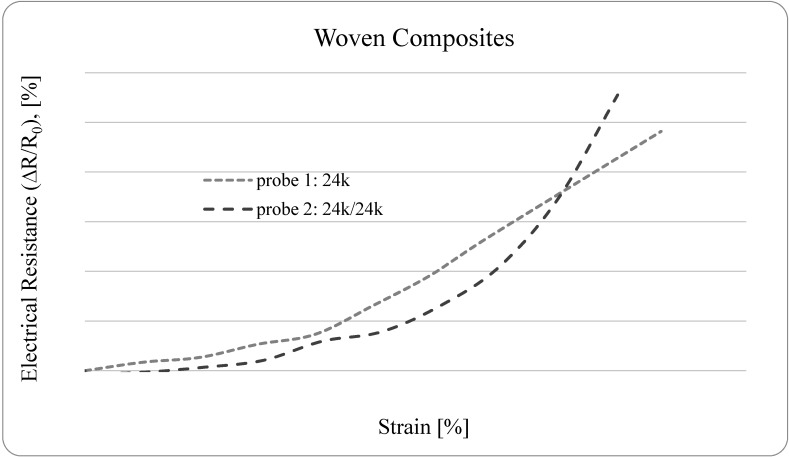
The relative electrical resistance *versus* strain of Textile Woven Epoxy Composites.

Based on these preliminary results, the composites reinforced with textile fabric were also tested at the dynamometer. These woven fabrics, easily produced by conventional textile weaving technologies, have specific arrangement that is well suited for both strengthening and monitoring of structures. Figure 7 presents the relative increase in electrical resistance (∆*R/R*_0_), in percentage, as function of the strain. The measured electrical resistance includes both volume and contact electrical resistances. The contact resistance of the connections between the measuring probes and the fibers, which was consistently minimized during the experiments, is assumed negligible.

As observed in Figure 7, the small strains cause an increase in the relative electrical resistance of the woven reinforced composites. Therefore, the fabric seems well suited to integrate a composite that is able to self-monitor the strain.

### 3.3. Laboratory Flexural Strength Loading Tests of Concrete Slabs

**Figure 8:**
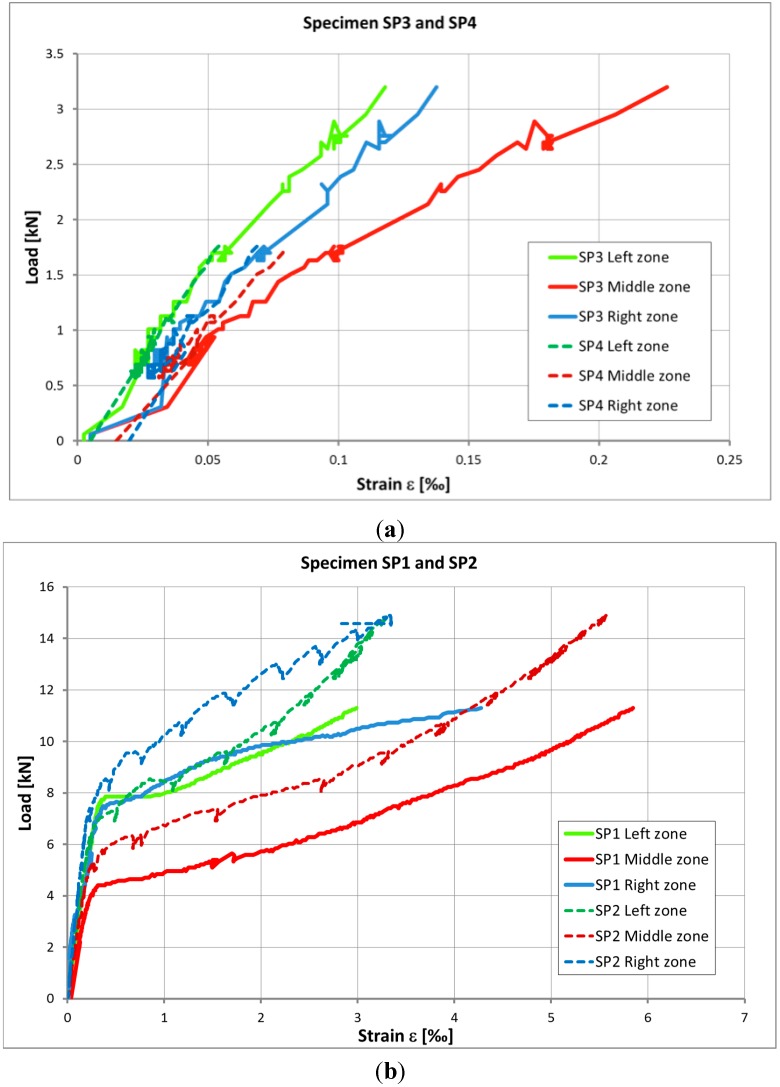
Strain-Load relationship for: (**a**) specimens SP3 and SP4 (without reinforcement) and (**b**) specimens SP1 and SP2 (with reinforcement).

Figure 8 presents the strain-load relationships given by the averaged data of the strain gauges placed on the left (2 strain gauges), middle (3 strain gauges), and right (2 strain gauges) zones of the probes. For all four specimens the elastic behavior of concrete (linear relationship) before rupture can clearly be identified.

**Figure 9:**
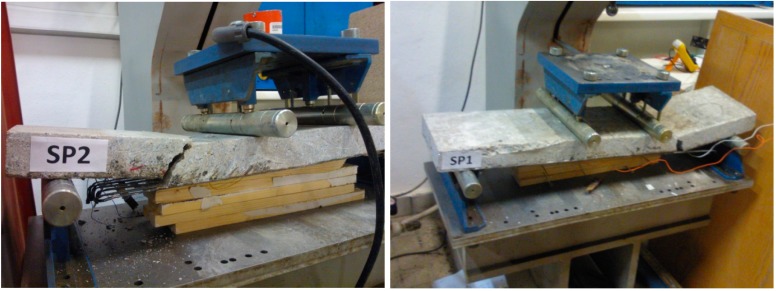
Damages and cracks for specimens SP2 and SP1.

Specimens SP3 and SP4 (plain concrete without strengthening) cracked directly after reaching the tensile strength of concrete. Specimens SP1 and SP2 (plain concrete strengthened by carbon tow composite) showed higher bearing capacity. The chart in Figure 8b shows the elastic part of the mechanical behavior of concrete and then the significant yielding part, after crack starts appearing, when fibers start to participate in carrying the tensile stress. As seen in Figure 9, for specimens SP1 and SP2 rupture suddenly appeared after delamination phenomena, which occurred for a significant strain, higher than 5‰.

**Figure 10:**
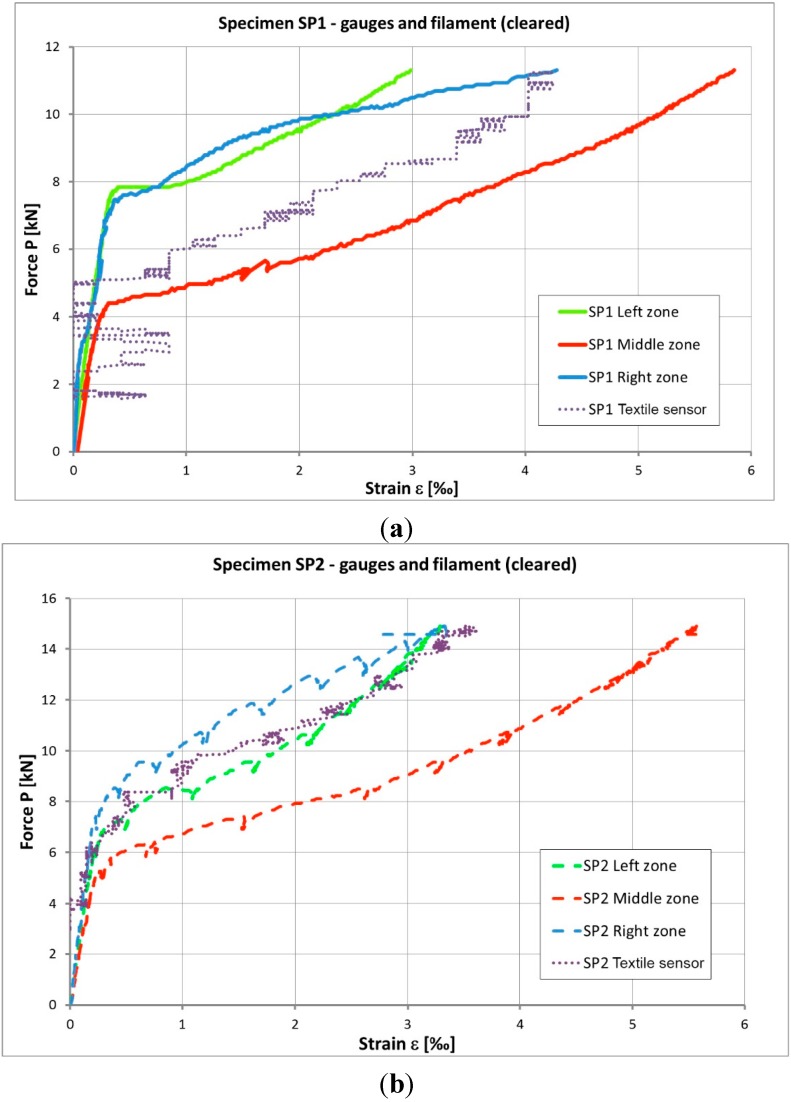
Strain—Load relationships for: (**a**) specimen SP1 and (**b**) specimen SP2.

Figure 10 shows the results obtained with the strain gauges and also the relative electrical resistance change of the carbon tow composite, after treatment, for specimens SP1 and SP2. Both measurements (gauges and fibers) present a similar path.

### 3.4. Laboratory Tests of Reinforced Concrete Beams

**Figure 11:**
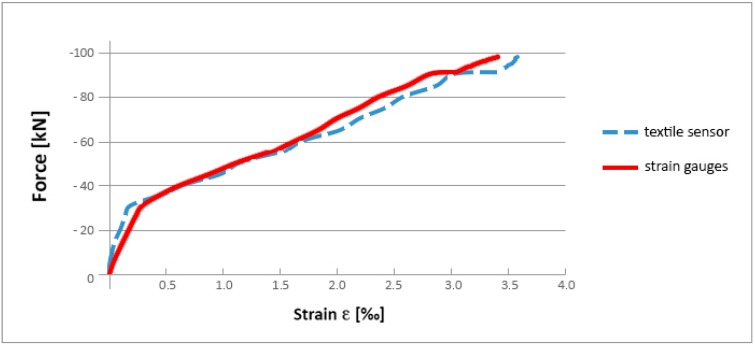
Comparison of averaged strain measured with textile sensor and with a set of nine conventional strain gauges.

Figure 11 presents the results obtained in the calibrating tests of the textile sensor on natural scale beams. As observed, the path of the curves describing changes of strain under increasing external load is almost the same. The differences between readings made by the textile sensor do not exceed 5% of those made by the strain gauges.

Results for textile sensor and strain gauges were recorded using a multichannel Wheatstone bridge joined with a microcomputer. As the textile sensor has electrical resistance lower than the common standard strain gauges, an adjustable resistor was inserted in series with it to increase their resistance and adjust it to the range of values used by the measurement system.

The main objective of this test is to find the gauge factor of the developed textile sensor and its stability over the whole range of textile elongation.

The gauge factor, *GF*, is defined as: *GF* = (∆*R/R*_0_)/*ε*. Where ∆*R/R*_0_ is the ratio of the change in electrical resistance of the textile caused by strain to its initial resistance and *ε* is the average strain measured with nine traditional strain gauges.

**Figure 12:**
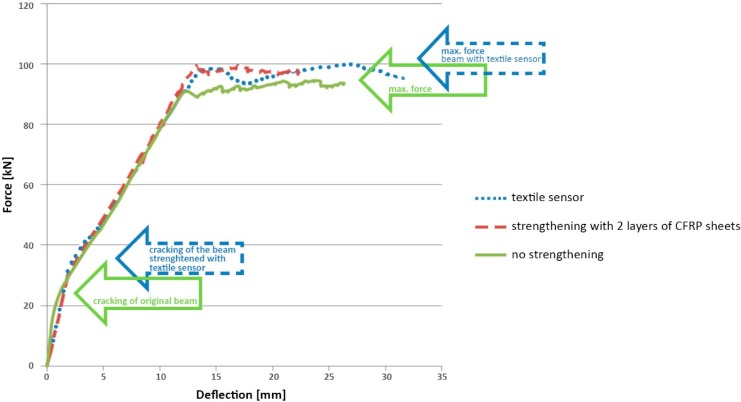
Relation between the external load and deflection for the three model beams: without strengthening, with carbon fiber reinforced polymer (CFRP) strengthening and with textile sensor strengthening.

For this textile sensor, the initial value of *GF* was calculated. It was about 9.5 with tendency to grow up to 11.5 near the failure strain. Therefore, an average value, equal to 10, was considered to compare the measured strains, shown in Figure 11 and Figure 12.

This test on a natural scale beam also allowed the verification of the effectiveness of the textile sensor for strengthening. The final loading capacity of the beam has increased 10%, which might be considered as not satisfactory. However it was noted, that such low efficiency is caused by too short anchorages of the textile strengthening. Nevertheless, the strengthening improved the behavior of the beam in all the phases of the work, and the first cracks appeared with a significantly higher force, as highlighted in Figure 12.

**Figure 13:**
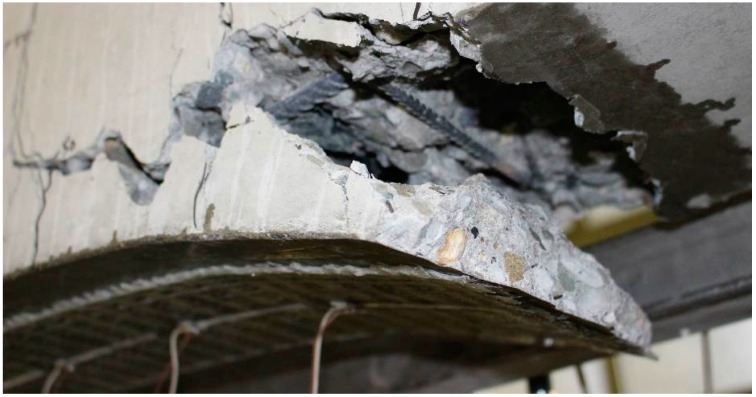
Failure of the RC beam strengthened with textile sensor.

Additional tests on RC beam strengthened with commercial CFRP mate (with the same length as the textile sensor) have shown identical mechanism of failure as in the one strengthened with textile sensor. In both cases, the failure of the beam by delamination of concrete occurs in zones of the anchorages of the strengthening, as it can be observed in Figure 13.

## 4. Conclusions

For all tested specimens at the dynamometer, the electrical resistance of the carbon fibers epoxy composites and of the woven epoxy composites changed with low strains, increasing with an increase of strain.

The carbon tow epoxy composite used for strengthening of concrete slabs, as well as the woven epoxy composite used for strengthening the natural scale beam, showed appropriated performance to increase the loading capacity of concrete elements, as well as to sense the deformation of the concrete elements subjected to external load. Indeed, the variation of the electrical resistance of the composites with the increasing strain presented similar path as the one registered by the strain gauges, giving promising results for further research. Future work should be focused on the development of a textile fabric for universal final application for both strengthening and self-sensing, and its calibration as a strain sensor, considering the temperature sensitivity of carbon fibers.

One may conclude that carbon tow normally used for strengthening the structures of buildings might behave as a sensor to detect small deformations (below 1%) of the concrete element. Moreover, by introducing some specific and simple designs on the arrangement of the continuous fibers that integrate the strengthening woven fabric, the self-sensing ability of the fabric might be improved. Some guidelines to develop the textile sensor may be established in order to increase its initial electrical resistance and to increase the change of electrical resistance with the strain, by:

- decreasing the number of filaments of the tow or
- increasing the length of the sensing fibers or
- introducing disruptions on the electrical pathway or
- crossing tows, defining contact zones between two sensing tows.

Moreover, such textile fabric for strengthening and sensing strain may be applied by techniques commonly used in Civil Engineering for the strengthening of all type of structures. Following these guidelines, strengthening fabrics made on carbon continuous fibers may be proposed as self-sensors for the health monitoring of structural elements. These textile fabrics are lightweight, low cost, multi-functional material, able to both strengthening and monitoring the health of structures of buildings.
